# Supplementary material for: Genomic mechanisms of climate adaptation in polyploid bioenergy switchgrass
Source: Nature. 2021 Jan 27;590(7846):438–44. doi: 10.1038/s41586-020-03127-1 (PMC7886653; doi:10.1038/s41586-020-03127-1)
Supplement: Supplementary file 1 — Examples of gating strategy in estimates of ploidy from flow cytometry. [file 41586_2020_3127_MOESM1_ESM.pdf]

natureresearch
https://doi.org/10.1038/s41586-020-03127-1

Supplementary information

Genomic mechanisms of climate adaptation in polyploid bioenergy switchgrass

In the format provided by the authors and unedited

a *Example of gating strategy for octoploids (8x)*

b *Example of gating strategy for tetraploids (4x)*

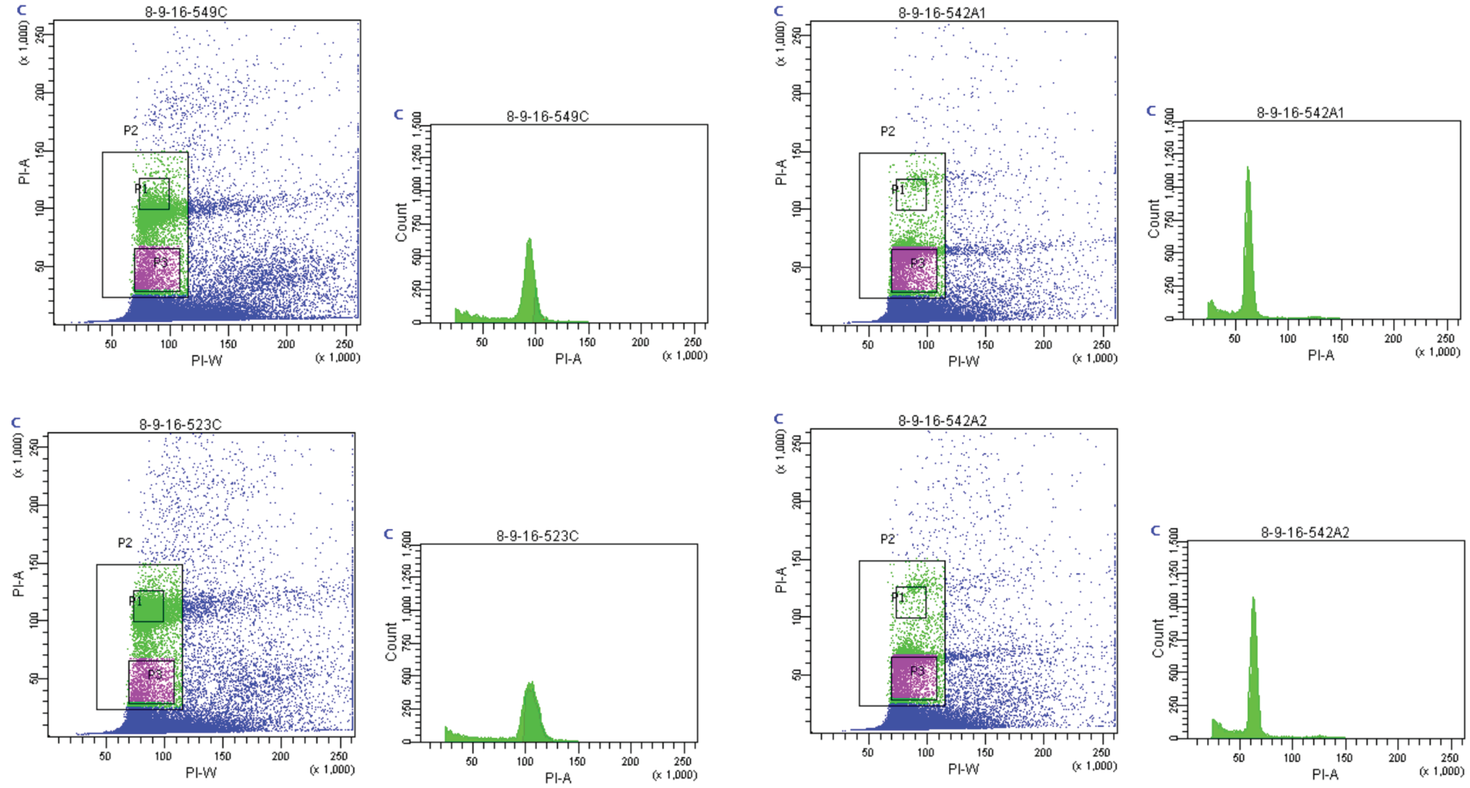

Supplementary Figure 1 I Gating strategy for determination of ploidy. Plots, generated in ICMB Flow Cytometry Lab, showing the observations overall, and those in the P1 and P3 gates. Those green and purple points (respectively) were used to determine ploidy. **a**, Histograms demonstrate clear peaks of ~100-120 for two representative 8x genotypes and **b**, ~60 for two representative 4x genotypes.
