# Supplementary material for: Genomic mechanisms of climate adaptation in polyploid bioenergy switchgrass
Source: Nature. 2021 Jan 27;590(7846):438–44. doi: 10.1038/s41586-020-03127-1 (PMC7886653; doi:10.1038/s41586-020-03127-1)
Supplement: Supplementary file 2 — Reporting Summary [file 41586_2020_3127_MOESM2_ESM.pdf]

nature research

Corresponding author(s): John Lovell

Last updated by author(s): Dec 10, 2020

Reporting Summary

Nature Research wishes to improve the reproducibility of the work that we publish. This form provides structure for consistency and transparency in reporting. For further information on Nature Research policies, see our Editorial Policies and the Editorial Policy Checklist.

Statistics

For all statistical analyses, confirm that the following items are present in the figure legend, table legend, main text, or Methods section.

n/a	Confirmed	
☐	☒	The exact sample size (*n*) for each experimental group/condition, given as a discrete number and unit of measurement
☒	☐	A statement on whether measurements were taken from distinct samples or whether the same sample was measured repeatedly
☐	☒	The statistical test(s) used AND whether they are one- or two-sided<br>*Only common tests should be described solely by name; describe more complex techniques in the Methods section.*
☐	☒	A description of all covariates tested
☐	☒	A description of any assumptions or corrections, such as tests of normality and adjustment for multiple comparisons
☐	☒	A full description of the statistical parameters including central tendency (e.g. means) or other basic estimates (e.g. regression coefficient) AND variation (e.g. standard deviation) or associated estimates of uncertainty (e.g. confidence intervals)
☐	☒	For null hypothesis testing, the test statistic (e.g. *F*, *t*, *r*) with confidence intervals, effect sizes, degrees of freedom and *P* value noted<br>*Give P values as exact values whenever suitable.*
☐	☒	For Bayesian analysis, information on the choice of priors and Markov chain Monte Carlo settings
☐	☒	For hierarchical and complex designs, identification of the appropriate level for tests and full reporting of outcomes
☐	☒	Estimates of effect sizes (e.g. Cohen's *d*, Pearson's *r*), indicating how they were calculated

Our web collection on statistics for biologists contains articles on many of the points above.

Software and code

Policy information about availability of computer code

Data collection	DNA and RNA data were collected via Illumina and PacBio internal routines. All phenotype data was collected and input manually using best practices for quantitative genetics data.
Data analysis	All data analysis was conducted through programs described in the methods. The majority of which was accomplished in the R environment for statistical computing. Other programs included: ancestry_hmm, vcftools, bcftools, samtools, varscan, PLINK, SHAPEIT, bwa-mem, Picard, GATK, MSMC, GSNAP, HTSeq, Dialign-TX, Gblocks, mafft, orthofinder, Repeatmasker, RepeatModeler, PASA, EXONERATE, Jellyfish, LTRHarvest, MECAT, BLAT, ARROW, and FlowJo. The GWAS pipeline developed here can be found on github (see code availability statement).

For manuscripts utilizing custom algorithms or software that are central to the research but not yet described in published literature, software must be made available to editors and reviewers. We strongly encourage code deposition in a community repository (e.g. GitHub). See the Nature Research guidelines for submitting code & software for further information.

Data

Policy information about availability of data

All manuscripts must include a data availability statement. This statement should provide the following information, where applicable:

- Accession codes, unique identifiers, or web links for publicly available datasets
- A list of figures that have associated raw data
- A description of any restrictions on data availability

SRA accession codes for all RNA and DNA sequencing libraries can be found in Supplemental Data 3 and 4 respectively. The v5 AP13 genome has be deposited at DDBJ/ENA/GenBank under the accession JABWAI000000000. The genome, gene and repeat annotations can also be downloaded directly from Phytozome: https://phytozome-next.jgi.doe.gov/info/Pvirgatum_v5_1. With the exception of map layers (Fig. 2a, Fig. 3a), which are publicly available from naturalearth.org, raw data

for all figures can be found in the source data file or associated tables in the extended data and supplementary material.

Field-specific reporting

Please select the one below that is the best fit for your research. If you are not sure, read the appropriate sections before making your selection.

☐ Life sciences ☐ Behavioural & social sciences ☒ Ecological, evolutionary & environmental sciences

For a reference copy of the document with all sections, see nature.com/documents/nr-reporting-summary-flat.pdf

Ecological, evolutionary & environmental sciences study design

All studies must disclose on these points even when the disclosure is negative.

Study description	We conducted quantitative analysis of phenotypes, collected in common gardens, and gene expression, collected in both common gardens and controlled conditions in the lab.
Research sample	Panicum virgatum (switchgrass) plants represent the entirety of the study design. Individual genotypes were clonally replicated and phenotyped in the field. Leaf and other tissue was assayed for gene expression. Replicated measures on a single individual plant were collapsed to a breeding value and never used as a unit of replication in any analyses.
Sampling strategy	Phenotyping was always conducted in a completely randomized design within blocks (common gardens).
Data collection	Field data were collected by a team of field technicians. The identities of the field techs always accompanied the measurements and care was taken to ensure that no systematic biases resulted from field technician factors.
Timing and spatial scale	Sample size was determined as a function of field experimental restrictions and sequencing cost. We sequenced 732 genotypes to maximize diversity within a limited budget. These plants were grown in as many sites as possible. For some sites, there was not enough space to grow all plants. In these cases, we chose plants that (a) represented the maximum genetic diversity and (b) had enough clonal replicates available.
Data exclusions	We discuss the libraries excluded in the methods. Some libraries were excluded due to poor sequencing quality or likely contamination.
Reproducibility	Plants were grown as clonal replicates. We opted for this approach (in lieu of full/half sib designs) to maximize repeatability: the exact same genotypes can be grown in other experiments.
Randomization	At each garden, planting was completely randomized in a single block.
Blinding	All field experiments were conducted using genotype identifiers that do not have an obvious connection to the location, name, etc. of each genotype. The anonymous 4- or 5-digit 'Library ID' was used for all statistical genomic analyses. It is impossible to conduct analyses blind of these identifiers, since all data is entered and output along with the IDs; however, we took care to use only these anonymous IDs and without direct reference to their biological names or context.

Did the study involve field work? ☒ Yes ☐ No

Field work, collection and transport

Field conditions	Field conditions were ambient at 10 common gardens over two years. Daily rainfall, temperature and soil conditions can be made available, but represent far too much data to place in this document. Summary climate data can be found in extended data figure 2.
Location	Here are the georeferenced coordinates of the 10 common gardens:<br>BRKG: 44.30680(lat), -96.67050(lon)<br>CLMB: 38.89690(lat), -92.21780(lon)<br>FRMI: 41.83671(lat), -88.23960(lon)<br>KBSM: 42.41962(lat), -85.37127(lon)<br>KING: 27.54986(lat), -97.88101(lon)<br>LINC: 41.15430(lat), -96.41530(lon)<br>OVTN: 32.30290(lat), -94.97940(lon)<br>PKLE: 30.38398(lat), -97.72938(lon)<br>STIL: 35.99115(lat), -97.04649(lon)<br>TMPL: 31.04338(lat), -97.34950(lon)
Access & import/export	All plant collections were conducted either from established agricultural gardens under the managers permission, or from collaborators under their own collecting permits.
Disturbance	Collections of natural habitats were conducted with the utmost care by professional botanists following protocols outlined in the collection permits. Common garden field sites were always constructed in previously disturbed or agricultural lands.

Reporting for specific materials, systems and methods

We require information from authors about some types of materials, experimental systems and methods used in many studies. Here, indicate whether each material, system or method listed is relevant to your study. If you are not sure if a list item applies to your research, read the appropriate section before selecting a response.

Materials & experimental systems

n/a	Involved in the study
☒	☐ Antibodies
☒	☐ Eukaryotic cell lines
☒	☐ Palaeontology and archaeology
☒	☐ Animals and other organisms
☒	☐ Human research participants
☒	☐ Clinical data
☒	☐ Dual use research of concern

Methods

n/a	Involved in the study
☒	☐ ChIP-seq
☐	☒ Flow cytometry
☒	☐ MRI-based neuroimaging

Flow Cytometry

Plots

Confirm that:

☒ The axis labels state the marker and fluorochrome used (e.g. CD4-FITC).

☒ The axis scales are clearly visible. Include numbers along axes only for bottom left plot of group (a 'group' is an analysis of identical markers).

☒ All plots are contour plots with outliers or pseudocolor plots.

☒ A numerical value for number of cells or percentage (with statistics) is provided.

Methodology

Sample preparation	200-300 mg of young leaf tissue was macerated in a petri dish with a razor blade and treated for 15 minutes with 1mL Cystain PI Absolute P nuclei extraction buffer (Sysmex Flow Cytometry) mixed with 1µL 2-mercaptoethanol. Samples were then filtered to isolate free nuclei with a CellTrics 30 µm filter (Sysmex) and treated for 20 minutes on wet ice with 2mL of Cystain PI Absolute P staining buffer (Sysmex), 12µL of propidium iodide and 6µL of RNase A
Instrument	LSRFortessa SORP Flow Cytometer (BD Biosciences)
Software	FlowJo software (BD Biosciences)
Cell population abundance	NA
Gating strategy	Samples were binned into three categories based upon the average units of fluorescence per nuclei. Ploidy level of the sample was considered 4X if the cell population had 40-80K units of fluorescence, 6X for 80-100K units and 8X for 100-140K units. The binning parameters were established with flow cytometry data from several P. virgatum accessions of known ploidy. T

☒ Tick this box to confirm that a figure exemplifying the gating strategy is provided in the Supplementary Information.
